# A Review of UK-Registered and Candidate Vaccines for Bovine Respiratory Disease

**DOI:** 10.3390/vaccines9121403

**Published:** 2021-11-27

**Authors:** Joanne L. Lemon, Michael J. McMenamy

**Affiliations:** 1 Sustainable Agri-Food and Sciences Division, Agri-Food and Bioscience Institute, Newforge Lane, Belfast BT9 5PX, UK; 2 Veterinary Sciences Division, Agri-Food and Bioscience Institute, Stormont, Belfast BT4 3SD, UK; michael.mcmenamy@afbini.gov.uk

**Keywords:** vaccination, pathogens, efficacy, non-microbial risks, candidate

## Abstract

Vaccination is widely regarded as a cornerstone in animal or herd health and infectious disease management. Nineteen vaccines against the major pathogens implicated in bovine respiratory disease are registered for use in the UK by the Veterinary Medicines Directorate (VMD). However, despite annual prophylactic vaccination, bovine respiratory disease is still conservatively estimated to cost the UK economy approximately £80 million per annum. This review examines the vaccine types available, discusses the surrounding literature and scientific rationale of the limitations and assesses the potential of novel vaccine technologies.

## 1. Introduction

Bovine respiratory disease complex (BRDC) is the principal cause of mortality in calves from 1–24 months of age across the world [1]. It has a significant impact on the global economy—the National Animal Disease Information Service (NADIS) estimates a cost to the UK alone of £80 million per annum, with over 1.9 million animals affected [2]. In the US this figure is estimated to be around $54.1 million, with over 1/5th of cattle affected in any given year [3]. Additionally, BRDC is a recurring problem in many other parts of the world [4,5,6,7] and is widespread across Europe [8,9,10,11]. 

Costs involved can be both direct and incidental—from mortality, weight loss or carcass blemish and subsequent reduced market price to additional labour expenditure, housing modifications and prophylactic or therapeutic treatments [3,12,13,14]. Respiratory disease can occur throughout the year, however BRDC is largely seasonal in nature with outbreaks occurring within one month of housing in autumn or early winter, thus vaccination usually occurs in late summer [15].

Although the focus of this paper is the bovine respiratory disease complex, multiple coinfections or superinfections from numerous respiratory pathogens lead to similarly critical complexes in many other species, such as in pigs [16] sheep [17,18] dogs (also known as kennel cough) [19] and cats [20] and follows the same disease course. Initially, the immunosupression arising from stressors and bovine viral diarrhoea virus (BVDV) infection increases vulnerability and thus the likelihood of viral infection. Viral infection has a dual effect on disease progression—first, there is direct damage to the airway epithelial layer and mucociliary escalator, thereby increasing susceptibility to secondary bacterial infection; secondly, the immunosuppressive nature of viral infection can lead to a decrease in the potency of immune responses, thereby potentially increasing opportunity for bacterial pathogens to be inhaled deeply into the lungs, causing lower respiratory tract (LRT) disease [21,22,23]. Consequently, immunity is further suppressed, potentially aiding invasion from opportunistic non-commensal bacteria [16,24,25,26]. This results in bovine respiratory disease which presents as calf pneumonia. This concludes in either calf death or reduced growth after recovery. 

Viral and bacterial microorganisms are the aetiological agents instigating BRD. Alongside, several non-microbial aspects have been identified as potential risk factors contributing to the risk of incidence of respiratory disease, discussed below. BRD is a multifaceted complex making it difficult to establish the exact contribution of each potential risk factor in decreasing resistance. However, all contributory risk factors act to elevate stress levels, reducing immunity and thus increase the susceptibility of cattle, particularly neonates [27]. Suitable alleviating mechanisms and infrastructures can reduce the severity and frequency of incidence of BRD: 

(1) *Housing*: Housing, more specifically ventilation and stocking density, is often cited as the largest non-microbial risk factor for the development of BRD in 0–3 month old calves [28].

(2) *Transport*: There is a strong link between transport and BRDC-related morbidity [29]. In terms of both distance and method, transport is acknowledged as being a major stressor for cattle and calves are at the highest risk of developing respiratory disease just after shipment [30]. Often BRDC is referred to as ‘shipping disease’ for this reason.

(3) *Weather*: Sudden and extreme temperature changes may have more of an impact on the risk of bovine respiratory disease developing than continually high or low temperatures [30,31]. However, evidence of this is inconsistent [32,33].

(4) *Farm management*: Many farm management and animal husbandry practices increase the risk of bovine respiratory disease developing including pre-movement activities (dehorning, castration, weaning), comingling, vaccination status and intensity of farming [34,35].

(5) *Genetics*: Various studies report Charolais, Simmental, Blonde d’Aquitaine and Aberdeen Angus bulls to have greater resistance to BRDC than other breeds [36]. Other authors have suggested that calves intended for use in the beef industry have a lower risk of developing BRD than those in the dairy industry, thought to be due to a greater microflora diversity and additional pathogen exposure through cattle markets [37]. Several groups are now using molecular techniques to identify genetically vulnerable animals [38,39,40].

The main contributory pathogens are detailed in Table 1, below. More recently, metagenomic analysis highlighted the involvement of up to 21 viruses in including bovine nidovirus, bovine parvovirus 3 and bovine rhinitis viruses [41]. Additionally it suggests the involvement of Influenza D virus—a newly identified virus, first detected in the UK in the winter of 2017 [42]. Frequently, the bacterial and viral pathogens associated with BRDC interact synergistically to enhance disease [43,44,45] although often the exact mechanisms remain unclear.

Due to the complex aetiology surrounding the establishment of BRD it is difficult to ascertain the exact contribution of each pathogen. However, it is recognised that, on a global scale, seropositivity rates against all viral and bacterial associated with BRD are high and can sometimes be up to 100% Europe [46,47,48,49,50]. Clinical disease is frequently most severe in calves under 6 months of age, even in those with maternal antibodies [51].

## 2. Currently Available Vaccines against BRD

Nineteen vaccines against BRD are registered for use in the UK by the Veterinary Medicine Directorate [52]. Eight vaccines designed to target the viral and bacterial pathogens of BRD are multi or polyvalent and thus designed to target several pathogens in one vaccine, while 11 are monovalent (Appendix A; Table A1 and Table A2). All vaccines available use whole virus, either modified live (attenuated) or inactivated, and all are administered by intramuscular, intranasal or subcutaneous routes. 

## 3. Limitations of Currently Available BRD Vaccines

Ineffective vaccines, declining employment in the agricultural sector and increasing awareness of antimicrobial resistance has led policymakers to shift the focus onto the development of superior, more efficacious vaccines as a major contribution in reducing the pressure to intensify on the farming sector. Although many vaccines against BRD are currently available on the UK market, they have limitations. Only a few of the vaccines have been registered as suitable for use in pregnant or lactating cows and all require refrigeration. Additionally, all come with a strong recommendation for a booster to advance immunity and none have been tested for maternal antibody interference [52]. Only eleven of the vaccines registered for use in the UK are multivalent and only four have been tested and deemed suitable for use alongside other veterinary treatments, frequently with those of the same manufacturer. However, multiple pathogens are considered threats during the neonatal stage and so it is impractical and ineffectual to have monovalent or incompatible medicines. Vaccination against BRD presents many challenges:

### 3.1. Age of Administration

A major challenge to the development of a successful vaccines for BRD is the age at which calves must be vaccinated [53]. Peak viral infection occurs upwards from 1 month so vaccination must take place in the first few weeks of life to allow immunity to develop [54,55]. However, evidence shows a calf’s immune system to be immature at this time, thought to be a carryover from the immunotolerant state induced during pregnancy [53,56,57]. To compound this problem, several essential farm management practices (discussed earlier) occur during this period increasing corticosteroid levels [30] and essential maternally-derived antibodies (MDA) may interfere with the development of any vaccine-induced immunity [54,58].

### 3.2. Route of Administration

The majority of currently approved vaccines against BRD are to be used parenterally (i.e., sub-cutaneously or intramuscularly) and these have been demonstrated as producing protective immune responses [59,60,61,62]. However, parenteral vaccines are invasive, require trained personnel for sterile administration and often cause a ‘depot effect’ at the local site of injection; in cattle this can lead to carcass scarring and thus reduced price [63]. More recently, epicutaneous vaccination using skin patches, a non-invasive, needle-free delivery route, has been investigated in mice against RSV with encouraging results [64].

It has also been hypothesised that it might be more rational to vaccinate at the initial site of pathogen entry—intranasally—thereby potentially preventing infection at source. Many more intranasal vaccines are getting approved and coming onto the market. Intranasal vaccination induces more localised and protective mucosal immunity through activation of nasal-associated lymphoid tissues (NALT). Although mucosal immunity can also be generated as a consequence of vaginal, anal and oral inoculation, intranasal delivery is preferable due to its many advantages (discussed in context with disadvantages in Table 2). In support of this, a study by Ellis et al. showed that intranasal administration of BRSV vaccines intended for parenteral use did not reduce the protective efficacy of the vaccines [65]. Additionally, Rossi et al. [66] demonstrated strong bronchoalveolar cell-mediated and antibody responses after a single intranasal delivery of a multivalent BRSV, BHV-1 and BPIV-3 vaccine.

### 3.3. Type of Vaccines Available:

#### 3.3.1. Modified-Live (MLV) Vaccines

Modified-live vaccines, also called attenuated vaccines, are those which employ live replicating whole pathogen that has been weakened in the laboratory. Attenuation of pathogenic strains can be obtained by modifying the molecular construction of the genome, using chemical mutagenesis, gene deletion or by extensive serial passaging in non-host cell culture or embryonated chick eggs. Chemical mutagenesis has also been coupled with low temperatures to develop a cold-adapted temperature-sensitive strain (ctss) of HRSV that can only replicate in the upper respiratory tract [67]. Only two diseases have been successfully eradicated across the globe—smallpox in humans [68] and rinderpest in cattle [69,70]—and both have been achieved using modified-live vaccines, thus illustrating their significant contribution to human and veterinary health. 

#### 3.3.2. Inactivated Vaccines

Inactivated vaccines, also known as killed vaccines, are those which do not contain any live replicating pathogenic material and cannot cause disease. For this reason they have a superior safety profile to their live counterparts [71] and are considered suitable for use in pregnant or lactating animals. The pathogenic agents are destroyed by heat, chemicals or radiation. Furthermore, inactivated vaccines do not require refrigeration and can be lyophilised for transport purposes [72].

#### 3.3.3. Immunogenicity of Modified-Live and Inactivated Vaccines

As attenuated vaccines broadly mimic the immune response garnered from a natural infection, they are universally recognised for producing stronger, longer lasting and more robust immune responses for many pathogens [72,73,74,75]. Furthermore, it is surmised that modified-live vaccines can initiate cellular responses in a way that inactivated vaccines are not reported as doing [76,77]. Several studies report the benefits of using modified-live vaccines in calves [78,79,80,81]. However, several studies now indicate that evidence on this is conflicting [81,82] and even if this were conclusive, often the immunogenicity advantages gained from MLV are offset by the increased safety risks posed, particularly in neonates. 

Conversely, the immune response garnered from using inactivated vaccines is considered by some as inadequate with suggestions that inactivated vaccines can effectively prime CD4+ T cells but encourage eosinophila [83] and others providing evidence that IFNγ expression is reduced [84]. A further study demonstrated a link between maternal vaccination for BVDV using inactivated vaccines and neonatal pancytopenia—a fatal autoimmune disease contracted from ingesting colostrum [85]. Further, although antibody titres can be high these are often found to be non-neutralising [86]. However, again, in contrast, several studies observed that using inactivated vaccines generated protection and they are at least as efficacious as using modified-live virus [83,87].

#### 3.3.4. Vaccine-Enhanced Disease

Of particular note for BRSV is the observation that vaccination could actually augment disease. This was first noted in 1967 after a failed vaccine trail using a formalin-inactivated RSV (FI-RSV) vaccine against HRSV [88] which led to investigations in cattle where a similar pathology was reported [89,90]. In this study, one group was vaccinated with a FI-BRSV vaccine while the other was sham-vaccinated. Both groups were challenged with live BRSV post-vaccination. No significant difference in gross lung lesions and in lung function was noted between the two groups, indicating the failure of the vaccine to provide any protective immunity. Further, although two groups were challenged with the same amount of BRSV, the sham vaccinated cohort demonstrated lower mean clinical scores [90] indicating disease exacerbation arising from vaccination. High titres of non-neutralising antibodies have also been observed, which can be associated with a high IgE titres and an allergic, inflammatory Th2-type response [91] and it is hypothesised that disease escalation is attributed to FI-RSV generation of low affinity antibodies [92] targeted at non-protective epitopes. Consequently, apprehension surrounds trials employing inactivated vaccines and scientists are cautious about developing candidate vaccines using inactivated antigen. Although antibody titres generated from vaccination are not always correlated with reduced disease, vaccination against any other pathogen implicated in BRD does not appear to have had such a detrimental effect [30].

### 3.4. Storage Conditions

Incorrect vaccine storage is frequently cited as a main reason for vaccine failure [93]. Correct storage conditions are essential for conserving the three-dimensional structure of antigens, and thus essential for vaccines to retain their potency [94]. However, reliable vaccine storage is often not controlled for in a field setting. Vaccines which could potentially remain immunogenic outside of the cold chain (i.e., not refrigerated) would be greatly beneficial to remote regions, vast farms or areas lacking sufficient infrastructure [95]. Recently a candidate nanoparticle RSV vaccine derived from an Sf9 insect cell line has been trialled showing that, once re-suspended, the vaccine can remain stable for < 60 days [96]. In further support of this, another group demonstrated that dry ice storage for up to 30 days did not detriment stability for a vaccine against East Coast fever—a tick-borne disease of cattle in Eastern and Central Africa with high mortality rates [97]. More recently a study into vaccines for tuberculosis showed that desiccation of liquid vaccine antigen increased thermostability outside of the cold-chain and produced a vaccine antigen more adaptable for mucosal use [98].

## 4. Practical Considerations for Vaccine Development

### 4.1. Antimicrobial Medicines

Metaphylaxis is a commonly employed tactic within farming, primarily utilised against mastitis in cattle, and dihydrostreptomycin, marbofloxacin or oxytetracycline are the most frequently detected antibiotics used in cattle [99]. Prophylactic treatment of calves with ceftiofur, rather than after the appearance of clinical signs was shown to reduce the incidence of BRD [100] further supporting this approach. However, this heavy reliance on antibiotics aggravated by the combination of agricultural intensification alongside ineffective vaccines has resulted in production systems coming under scrutiny as a source of escalating antimicrobial resistance. Globally, antimicrobial consumption in animal production is expected to increase by almost 70% by 2030 [101] despite comprehensive UK government efforts to curtail usage [102]. 

### 4.2. EU Regulations

Regulatory requirements for registering a veterinary vaccine within the EU are less stringent than those necessary to register a human vaccine; a process which is regulated by the Veterinary Medicines Directorate [103,104]. The legislative requirements are laid down in 2009/9/EC and/or by the World Organisation for Animal Health (OIE). Vaccine development is geared towards reducing animal use, with current investigations into in vitro potency assays [105] and *in silico* systems vaccinology [106].

Council directive 81/852/EEC and the European Pharmacopoeia further detail requirements, stating that results from laboratory efficacy trials should be supplemented with data from field efficacy trials [107]. However, when laboratory efficacy cannot be supported by field trials, in vitro data alone may be acceptable; this can particularly relevant for diseases which lack suitable experimental infection models (i.e., BRSV), with diseases that are caused by more than one aetiological agent or in certain diseases where environmental factors play a major role in disease development. The development of BRDC can be applied to any of these criteria [108]. Only two parameters need to be measured: clinical picture (mortality, morbidity, lesions, weigh) and the serological response, but evidence shows cell-mediated immunity to play an important role against bovine respiratory disease [109,110].

### 4.3. Veterinary Adjuvants

**Figure 1:**
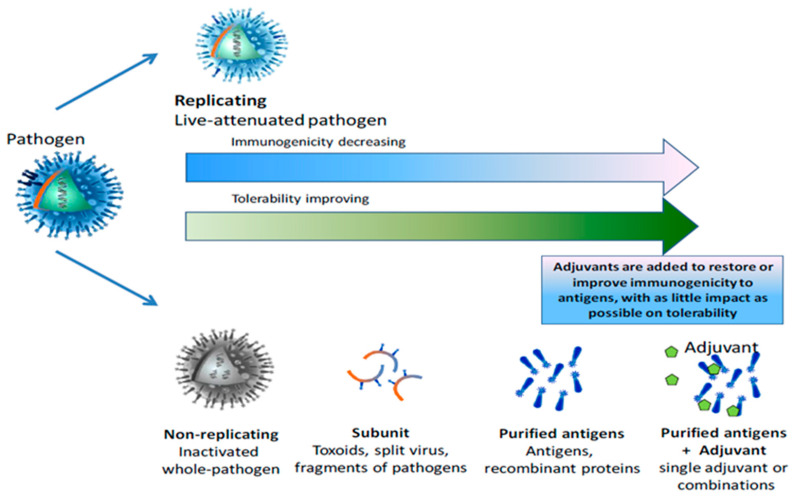
Vaccine antigen type consequence on vaccine safety and immunogenicity.

Vaccines which are poorly immunogenic often require an additional stimulant as a component to augment efficacy. These can potentially reduce vaccine antigen loads or administration frequency [111] thus reducing production costs. Removal of the pathogenic fragments of BRSV to leave only purified antigens (i.e., a sub-unit vaccine) will increase the safety profile of the vaccine, increasing the tolerability. However, this can also lead to a reduction in the immunogenicity and the vaccine-induced immune response generated [112]. This balance between immunogenicity and safety is represented in Figure 1. Unlike human medicine, several adjuvants are registered for use in animal vaccines and all currently available vaccines employ aluminium compounds or variants. Despite its potent induction of cell-mediated immunity, the use of Complete or Incomplete Freund’s Adjuvant (CFA/IFA) is strictly controlled in veterinary vaccines due to toxicity and the induction of painful side effects [113,114]. Montanide is emerging as a novel veterinary adjuvant suitable for use in cattle due to the higher lymphoproliferative and antibody responses observed in vaccines in which it is incorporated [115,116]. The area of research on adjuvants is expanding quickly and other adjuvants under investigation for use in cattle vaccines include IL-18 [117] poly(diaminosulfide) nanoparticles [118] and bovine IL17A [119].

### 4.4. Vaccination Regimes

Current vaccination regimes rely on a prime/boost regimen to obtain the greatest vaccine-induced immune response [120] and traditionally, boosting occurred with a homologous vaccine. Hill et al. demonstrated that intranasal vaccination of 3–6 week old calves using a modified-live BHV-1 vaccine followed by a booster with the same vaccine produced significantly higher IgA titres, reduced viral shedding and resulted in the least weight loss than a second vaccination with a heterologous (sub-cutaneous) vaccine [121]. However, other studies suggest that boosting with a heterologous vaccine can have a more beneficial effect as different arms of the immune system can be induced [122]. Important considerations are the vaccine antigen type [123] and the route of administration of the priming or boosting vaccine [124]. Due to the age at which BRD can develop and by extension the age at which vaccination is essential, induction of protective immunity following a single vaccine dose is highly desirable. Single dose vaccination has shown promising results in calves with MDA, using a modified live or recombinant BRSV vaccine administered intranasally or intramuscularly [62,125,126]. However, often little change is detected in virus neutralising antibodies (VNA) titres indicating that single inoculations may only be efficient at priming cell-mediated immunity [127]. 

### 4.5. Pre-Partum Vaccination

Pre-partum vaccination of a heifer or cow is a farm management strategy successfully used to provide immunity for neonates, primarily against enteric pathogens such as *Escherichia coli (E. coli)*, rotavirus or coronavirus [128,129]. Few report findings of maternal vaccination against BRD. However, Dudek et al. observed that maternal vaccination with a multivalent inactivated BRSV/BPIV-3/*M. haemolytica* vaccine, boosted colostrum immunoglobulin levels and led to increase in blood antibody titres in calves [130]. Additionally, it was reported that pre-partum vaccination positively impacted upon the antibody levels for BRSV, BHV-1, BVDV and BPIV-3. However, it was not considered significant enough to negate recommended neonatal vaccination schedules [131]. 

## 5. Candidate BRD Vaccines

Control strategies surrounding bovine respiratory disease concentrate on seasonal prophylactic antibiotic treatment and the administration of vaccines. Thus far these strategies have failed to prevent disease, with an estimated 1.9 million animals in the UK still affected by bovine respiratory disease annually. With increasing pressures on farms to intensify production alongside more rigid control on antibiotic use, research has focused on developing more efficacious, next generation vaccines.

### 5.1. Peptide Vaccines

There are currently no commercially available peptide vaccines for use in the veterinary field, despite the potential advantages in terms of ease of manufacture and flexibility. However, success has been reported for several candidate peptide vaccines and currently there is much interest in their use in human medicine, particularly against cancers [132], Human Immunodeficiency Virus [133] and diabetes [134]. In farm animals, Foot and Mouth Disease virus (FMDV) seems to command the largest share of research into peptide vaccines, in part due its status as a Specified Animal Pathogen Order (SAPO) level 4 infectious agent and of the economic impact of an outbreak [135,136]. Greenwood et al. describe using inert nanobeads conjugated with peptides from FMDV in sheep with higher antibody titres, increased TNFα, IFNγ, IL-6 and greater T cell proliferation observed in immunised animals [137]. Vaccines based on epitopes of the FMDV surface protein VP1 have previously shown promise in swine [138] and Zhang et al. recently reported success with a peptide vaccine against FMDV detailing 100% protection after prime/boost vaccination and challenge in cattle [139]. Bastien et al. reported a reduction in pathological lesions, compared to unvaccinated controls, when calves were immunised with a peptide encompassing BRSV G_174–187_ [140]. This had previously been shown to confer protection in mice [141]. Additionally, intraperitoneal immunisation of mice with a recombinant protein comprising HRSV G_130–230_ coupled to streptococcal G protein protected mice from upper and lower respiratory tract infection [142]. However, despite the induction of high antibody titres in these studies, they were non-neutralising; a result repeated in a later study [143] implying that peptide vaccines provide protection by means other than a humoral response. 

### 5.2. Immune Stimulatory Complexes (ISCOMs)

ISCOMS are comprised of viral glycoproteins, cholesterol, phospholipids and non-toxic saponins from the bark of the Quillaja saponin tree (*Quillaja saponaria*). Tested using the surface antigens of HRSV and BRSV in guinea pigs [144] they have shown promise when used against BRSV in young calves with maternal antibodies [145]. Additionally, a bovine herpesvirus ISCOM vaccine demonstrated a high degree of immunogenicity when compared to the same BHV-1 proteins administered alone [146] and an ISCOM BVDV vaccine, which had previously been shown as protective in sheep, produced high serum neutralising titres when administered subcutaneously to calves [147]. 

### 5.3. Virus-Like Particles (VLP)

Virus-like particles (also known as pseudoviral particles) functionally and structurally resemble viruses and present viral antigens in a conformation more akin to a virion. As such, there is potential for lower antigenic doses to be used, reducing the cost of the vaccine—an important consideration in veterinary medicine [148]. As they do not contain genetic material they are non-infectious, non-replicating and safer than killed or attentuated vaccines. Although there is a lack of literature on VLPs for BRD, an early study demonstrated protection from clinical disease, significantly reduced leukopenia and led to the induction of cell-mediated and humoral immune responses when calves were immunised with a VLP containing BVDV 1b E2 glycoprotein antigens [149].

### 5.4. Nanoparticle Vaccines

To protect peptides from proteolytic degradation by cytosolic aminopeptidases they require formulation with an additional component such as carrier proteins or nanoparticles. Nanoparticles can be relatively inexpensive to manufacture, biodegradable non-toxic and can augment the immunogenicity of subunit vaccines, leading to a potential decrease in antigen load or administration frequency. Further benefits include controlled antigen release, providing protection to antigen in the unfavourable conditions provided by the respiratory tract and the potential removal of the cold chain necessary for vaccine storage [150]. Circular nanoring structures derived from a recombinant N protein of HRSV were used as a novel candidate vaccine against BRSV in young calves, with encouraging results [61]. A PLGA nanoparticle vaccine for BPIV-3 demonstrated higher IgA and serum IgG responses than those generated from a commercial vaccine, although there was no detection of any cell-mediated immunity [151]. Vaccines based on nanoparticles have also been associated with a reduced need for therapeutic intervention [152] and a recent review highlighted the benefits gained from using natural nanoparticles in livestock vaccines from a One Health perspective [153]. 

### 5.5. DNA Vaccines

DNA vaccines evolved in the 1990s [154] and have since shown success against many veterinary pathogens [155,156,157]. The principal behind a DNA vaccine is that sequence encoding a protein of interest from the pathogen involved will be ligated into bacterial plasmid DNA, which is transfected in vitro and transferred to a host for in vivo transcription to subsequently induce an immune response [158]. Intradermal vaccination using a DNA vaccine which expressed the E2 glycoprotein of BVDV resulted in strong BVDV-specific T-cell proliferation and a significant rise in antibody titres [159]. An early study of an intramuscular DNA vaccine against Influenza A observed proficient stimulation of T and B cell responses, indicating an ability to cross-present antigen [160]. However, only some have proved promising for neonatal vaccination against BRD in young calves with maternally-derived antibodies [161]—many have only been tested in seronegative calves [162]. Furthermore, B cell responses have been observed as being slower to develop and ultimately lower in VNA titres than those from natural infection [163] or using a vectored vaccine [163]. More recently, a decrease in clinical disease and viral shedding in conjunction with an increased IFNƴ and IgG response was noted after vaccination with a BHV-1 DNA vaccine [164]. With DNA vaccines, unlike protein-based vaccines, no mis-folding can occur. Additionally, when the production, storage, stability and safety advantages are taken into consideration alongside their proven ability to elicit both a cytotoxic and humoral response, it is clear this is an area worth researching more [165].

### 5.6. Messenger RNA (mRNA) Vaccines

Messenger RNA vaccines are closely related to DNA vaccines with two major differences—firstly, mRNA does not need to be processed through the nucleus but instead is released directly into the cytoplasm for transcription and secondly, the need for effective transfection is negated [166]. Essentially, mRNA vaccines remove an additional molecular step needed by DNA vaccines to accelerate the generation of vaccine-induced immunity. The only approved mRNA vaccines on the market at present are against SARS-CoV-2 infection, responsible for the Covid 19 pandemic [167] and mRNA vaccines have continued to show promise in clinical trials for Zika virus [168], Influenza A [169] and rabies in humans [170]. The absence of published data relating to the use of mRNA vaccines in farm animals, and more specifically against the pathogens implicated in BRDC, serves to highlight the newness of this area of research. 

### 5.7. Viral Vectors

Viral vaccine vectors have been shown to capably induce robust cell mediated and humoral responses against *Mannheimia haemolytica* [171], BRSV [163], BHV-1 [172] and BVDV [173]. Unlike many other subunit vaccines often they do not require an adjuvant to boost immunity, instead relying on the inherent capability of a virus to enter and replicate in a cell. Poxvirus vectors show promise for multivalent vaccine use in cattle, due to the size of exogenous genes which can be accepted and they enjoy a superior safety profile due to their avian-restricted cytoplasmic replication. Vaccination with a recombinant modified Vaccinia virus Ankara (MVA) has shown much promise with BRSV [174] and its replication-deficiency makes it all the more suitable for use in the field. Importantly, potential disease-causing immunopathological reactions such as elevated IgE titres and eosinophil influx have not been observed in MVA vaccinated calves, post BRSV challenge [175]. Additionally, plant based vaccines are gaining prominence [176,177] and have shown efficacy when tested against BVDV [178]. Bovine respiratory pathogens are often used as vectors for other bovine respiratory pathogens—bovine herpesvirus-type 1 (BHV-1) has been successfully used against BRSV in calves reducing clinical signs, pneumonic lesions and viral loads despite low antibody titres [179] and a recombinant *Pasteurella multocida* vector has been shown to provide protection against *Mannheimia haemolytica* [180]. The majority of candidate vaccines using bovine respiratory pathogens as viral vectors focus on bovine RSV—as the model of BRSV disease in calves mirrors that of RSV infection in infants, this can be considered a reflection of the medical urgency for a human RSV vaccine. 

## 6. Conclusions

Bovine respiratory disease is a major threat to dairy and beef farming on a global scale and is the leading cause of mortality and morbidity in cattle over 1 month of age [181]. Currently, the commercially available vaccines against BRD are limited in their efficacy, as evidenced by the development of clinical disease regardless of vaccination. This is despite advances in understanding pathogenesis, aetiological agents, vaccine technologies and biosecurity measures [3]. This limited efficacy may be due to a combination inappropriate administration routes, unsuitable vaccine storage and the challenges of inoculating in young calves. Further, major obstacles in successful vaccine design can come from a combination of host immunity factors, pathogen characteristics and necessary on-farm practices. 

However, on a global scale the population is projected to grow exponentially with an estimated 70–100% increase in food production required to satisfy this growth [182]. To compound the problem of food security from rising demand, employment in agriculture has been on the decline since the 1900s [183,184]. The demand for additional food from fewer resources is a problem intrinsically linked to socio-economics, climate change, agriculture and resource or land use [185].

The UK relies heavily on agriculture as a source of income for the economy—in 2020 the total income from farming was estimated to be £4119 million [186]. Using Northern Ireland as an example, agriculture accounts for 1.7% of Gross Value Added (GVA), approximately 3 times above the UK average, and has been identified as a key future source of economic development in the ‘Going for Growth’ agri-food strategy [187]. However, the intensification necessary to realise this drive for economic growth has resulted in increasing farm size against a backdrop of decreasing farm numbers [188] and this intensification in productivity may lead to an increasing vulnerability to infectious disease. Thus, alongside increasing pressure on farms to intensify production and more rigid control on antibiotic use, research continues to focus on developing more efficacious, next generation vaccines [189].

## Figures and Tables

**Table 1:** Common viral and bacterial pathogens implicated in BRDC.

Viruses	Bacteria	Mycoplasma spp.
Bovine herpesvirus-1 (BHV-1)	*Mannheimia (Pasteurella) haemolytica*	*Mycoplasma bovis*
Bovine respiratory syncytial virus (BRSV)	*Pasteurella multocida*	*Ureaplasma* spp.
Bovine viral diarrhoea virus (BVDV)	*Histophilus (Haemophilus) somni*	
Bovine parainfluenza virus type-3 (BPIV-3)		
Bovine adenovirus (BAV)		
Bovine coronavirus (BCoV)		

**Table 2:** Main advantages and disadvantages of intranasal vaccination in cattle.

**Advantages**
More neutral pH and lower levels of enzymatic activity than digestive tract
Prime neonatal calf in the presence of MDA
Needle-free/non-invasive
Induction of systemic and mucosal immunity
User-friendly (potential use in herds/developing world/remote farms)
**Disadvantages**
Rapid clearance of low affinity antigens
Potential antigen loss during inoculation (impact on cost)
Inefficient uptake
Lack of compatible adjuvants for mucosal vaccines

## Data Availability

The data presented in this study are collected from the cited literature.

## Appendix A

**Table A1:** Monovalent vaccines for bovine respiratory disease.

Name:	**Bovalto Pastobov (2007; Vm 08327/4173)**
Multivalent:	N—*Mannheimia haemolytica* (formerly *Pasteurella haemolytica*)
Antigen:	Type A1
Recommended age:	Minimum 4 weeks, booster 28 days later
Route of administration:	Sub-cutaneous or intramuscular
Adjuvant/excipients:	Aluminium hydroxide (4.2 mg), thiomersal, salts, water
Name:	**Bovela lyophilisate (EU/2/14/176/001-016)**
Multivalent:	N—BVDV (1—non cyto KE-9; 2—non cyto NY-93)
Antigen:	Modified live (both strains)
Recommended age:	3 months +
Route of administration:	Intramuscular
Adjuvant/excipients:	Not detailed
Name:	**Bovilis BVDV suspension (1999; Vm06376/4025)**
Multivalent:	N—BVDV 1 (non cyto C-86)
Antigen:	Inactivated
Recommended age:	8 months
Route of administration:	Intramuscular
Adjuvant/excipients:	Aluminium 3+ (Alum hydroxide) 6–9 mg
Name:	**BOVILIS IBR marker inac (2006; vm06376/4053)**
Multivalent:	N—BHV-1 (GK/D gE negative strain)
Antigen:	Inactivated
Recommended age:	3 months onwards
Route of administration:	Intramuscular
Adjuvant/excipients:	Alum 3+ (6.0–8.8 mg) formaldehyde 0.6–1 mg, trometamol
Name:	**BOVILIS IBR MARKER LIVE (2006, vm06376/4032)**
Multivalent:	N—BHV-1 (GK/D strain, gE negative)
Antigen:	Live
Recommended age:	2 wk (Intranasal) 12 wk (Intramuscular)
Route of administration:	Intranasal or Intramuscular
Adjuvant/excipients:	Sorbitol, monosodium glutamate; glycine
Name:	**HIPRABOVIS IBR marker live (2011; eu/2/10/114/001)**
Multivalent:	N—BHV-1 (gE deleted; Ceddel strain)
Antigen:	Live
Recommended age:	12 wk +
Route of administration:	Intramuscular
Adjuvant/excipients:	Not disclosed
Name:	**NASYM (EU/2/19/241/001-004**
Multivalent:	N—BRSV (LYM-56)
Antigen:	Live
Recommended age:	9d (Intranasal) 10w (intramuscular)
Route of administration:	Intranasal OR Intramuscular
Adjuvant/excipients:	Not disclosed
Name:	**RISPOVAL IBR MARKER (VM42058/4127, 1999)**
Multivalent:	N—BHV-1 (gE deleted, Difivac Strain)
Antigen:	Inactivated
Recommended age:	2w–3 m (if later than use 3rd booster)
Route of administration:	Sub cut Sub-cutaneous
Adjuvant/excipients:	QUIL A (0.25 MG) ALUM HYDROX (14–24 MG)
Name:	**Rispoval Pateurella (VM42058/4128, 1999)**
Multivalent:	N—Man.H type A1 (NL 1009 strain)
Antigen:	Inactivated
Recommended age:	12 wk +
Route of administration:	Intramuscular
Adjuvant/excipients:	Liquid paraffin and alum hydrox
Name:	**Rispoval RS (vm 42058/4129, 2005)**
Multivalent:	N—BRSV (RB94)
Antigen:	Live attenuated
Recommended age:	7d–4m (If 7d then 3rd booster to counteract MDA)
Route of administration:	Intramuscular
Adjuvant/excipients:	Not detailed
Name:	**Tracherine (VM42058/4156; 2010)**
Multivalent:	N—IBR (RBL106)
Antigen:	Live attenuated
Recommended age:	3w (10 wk ideally for reduced MDA interference)
Route of administration:	Intranasal
Adjuvant/excipients:	Not detailed

**Table A2:** Poly/Multivalent vaccines for bovine respiratory disease.

Name:	**Bovalto Respi 3 (2016) (Vm 08327/4273)**
Multivalent:	Y—BRSV (BiO-24) and BPIV-3 (BiO-23) Man.H (A1)
Antigen:	All inactivated
Recommended age:	In cattle devoid of MDA (~6 months)
Route of administration:	Sub-cutaneous
Adjuvant/excipients:	AH (8.0 mg) Quil A (0.4 mg) Thiomersal (0.2 mg) Formaldehyde (1.0 mg)
Name:	**Bovalto Respi 4 (2016; Vm 08327/4274)**
Multivalent:	Y—BRSV (BiO24) BBPIV-3V (Bio23) Man H (A1) BVDVV (Bio25)
Antigen:	All inactivated
Recommended age:	2 wk+
Route of administration:	Sub-cutaneous
Adjuvant/excipients:	AH (8.0 mg) Quil A (0.4 mg) Thiomersal (0.2 mg) Formaldehyde (1.0 mg)
Name:	**Bovalto Respi Intranasal (2018; Vm 08327/4289)**
Multivalent:	Y—BPIV-3 (Bio23/A) BRSV (Bio24/A)
Antigen:	Modified live (both)
Recommended age:	10 day +
Route of administration:	Intranasal
Adjuvant/excipients:	Not detailed
Name:	**Bovilis bovipast rsp (1999; Vm 01708/4458)**
Multivalent:	Y—BRSV (ev908) BPIV-3 (sf-4 reisinger) Man.H A1 (cross reactive to A6)
Antigen:	All inactivated
Recommended age:	~2 wks of age, prior to housing
Route of administration:	Sub-cutaneous
Adjuvant/excipients:	Alum hydr (37.5 mg) Quil A (0.625 mg) Thiomersal (0.032–0.58 mg)
Name:	**IMURESP-RP (vm 42058/4072 2005)**
Multivalent:	Y—BPIV-3 (ts RLB103) IBR (ts RLB106)
Antigen:	Live attenuated
Recommended age:	3–10 wk but 10+ wk preferred
Route of administration:	Intranasal
Adjuvant/excipients:	Not disclosed
Name:	**Rispoval 3 (vm 42058/4124; 2005)**
Multivalent:	Y—BPIV-3 (RLB103) BRSV (375) BVDVV (type 1 5960 c + 6309 n-c)
Antigen:	Modified live (BPIV-3, BRSV) inactivated BVDV
Recommended age:	12 wk+
Route of administration:	Intramuscular
Adjuvant/excipients:	Alhydrogel 2% 0.8 mL (equiv. of 24.36 mg alum hydroxide)
Name:	**Rispoval 4 (VM42058/4125, 2001)**
Multivalent:	Y—As above (17) including IBR/BHV-1
Antigen:	Inactivated
Recommended age:	12 wk +
Route of administration:	Intramuscular
Adjuvant/excipients:	Not detailed
Name:	**Rispoval RS + BPIV-3 (VM42058/4130)**
Multivalent:	Y—BRSV (375) and BPIV-3 (ts RLB103)
Antigen:	Modified Live
Recommended age:	9 day +
Route of administration:	Intranasal
Adjuvant/excipient:	Not disclosed

ts = temperature sensitive; c = cytopathic, n-c = non-cytopathic.
